# Biosynthesis of Silver Nanoparticles and Exploring Their Potential of Reducing the Contamination of the In Vitro Culture Media and Inducing the Callus Growth of *Rumex nervosus* Explants

**DOI:** 10.3390/molecules28093666

**Published:** 2023-04-23

**Authors:** Norah S. Alfarraj, Mohamed Tarroum, Fahad Al-Qurainy, Mohammad Nadeem, Salim Khan, Abdalrhaman M. Salih, Hassan O. Shaikhaldein, Abdulrahman Al-Hashimi, Saleh Alansi, Kahkashan Perveen

**Affiliations:** Department of Botany and Microbiology, College of Science, King Saud University, Riyadh 11451, Saudi Arabia

**Keywords:** biosynthesis, silver nanoparticles (AgNPs), microbial contaminations, molecular markers

## Abstract

Among biological methods, green synthesis of the nanomaterials using plant extracts was shown to be an environmentally friendly, economical, and simple approach. In the current study, the biogenic synthesis of silver nanoparticles (AgNPs) was achieved using the leaf extract of *Hibiscus tiliaceus*, in order to prevent the contamination of the tissue culture media and induce callus growth. The nanostructures of the fabricated AgNPs were characterized using UV–visible spectroscopy, Fourier transform infra-red spectra (FTIR), X-ray diffraction (XRD), transmission electron microscopy (TEM), zeta size, and zeta potential techniques. Our results indicate that The UV–vis spectrum of AgNPs exhibited an absorption band at 415 nm. The FTIR analysis identified the functional groups which could involve in the reduction of silver ions to AgNPs, this was also confirmed by the (hkl) diffraction peaks in the XRD diffractogram. Moreover, the TEM analysis showed a spherical nanoparticle with a size ranging from 21 and 26 nm. Thereafter, the potential antibacterial and antifungal activity of the biogenic AgNPs was evaluated against *Bacillus pumilus* and *Alternaria alternata* which were isolated from the in vitro culture media and identified based on 16S rDNA and ITS rDNA sequences, respectively. The results showed that the AgNPs significantly inhibited the growth of *Alternaria alternata* and *Bacillus pumilus* at all applied concentrations (5, 10, 20 and 40 mg/L). Compared to the control more fungal radial growth reduction (42.59%,) and bacterial inhibition (98.12%) were registered in the plates containing high doses of AgNPs (40 mg/L). Using *Rumex nervosus* explants, the biosynthesized AgNPs were tested for their impact to promote callus growth. The obtained results showed a significant effect of AgNPs on callus fresh weight at all applied doses. Moreover, AgNPs treatments showed a polymorphism of 12.5% which was detected by RAPD markers. In summary, the results revealed that AgNPs (40 mg/L) can be effectively added to the in vitro culture media for reducing microbial contamination and improving callus growth while greatly maintaining its genetic stability.

## 1. Introduction

Nanotechnology is now widely seen as having the potential to bring benefits in various areas such as drug development, water decontamination, information and communication technologies, and the production of stronger and lighter materials. Nanotechnologies implicate the fabrication of materials at the nanometer scale, either by extrapolating from groups of single atoms or by refining or reducing bulk materials [1]. Nanoparticles are commonly known as particles with a maximum size of 100 nm showing different characteristics in comparison with the larger particles in the bulk material. The new properties of the nanoparticles are owing to the differences in specific properties, such as the size and the morphology of the particles [2]. The nanoparticles were fabricated and stabilized through different methods such as chemical, physical, and biological synthesis. However, the search for a suitable method for nanoparticle fabrication is the primary concern for researchers worldwide [3]. The biological way for nanoparticle synthesis is the preferable method since the distribution control of the obtained nanoparticles is more successful than the other approaches. Moreover, no harmful effect is accompanied by the biological synthesis of the nanoparticles [4]. As nano-factories, the potential of various microbes and plant biomasses were earlier used for the synthesis of nanometals. The plant extract contains various molecules from proteins to low molecular weight compounds such as terpenoids, alkaloids, amino acids, alcoholic compounds, polyphenols glutathiones, polysaccharides, antioxidants, organic acids, and the quinones, which were registered to play an important role in the biosynthesis, the reduction and the stability of the nanoparticles [5]. However, owing to the difference in concentrations of the bioactive molecules and the phytochemicals compounds as a result of seasonal or climatic changes, which affect their synthetic procedures, the nanoparticles may not be fabricated through the biosynthetic approach due to the requirement of a strong reducing agent [6]. The biosynthesis of nanoparticles using the plant extract has been recorded in many plant species [7,8,9]. Using leaf and flower extracts of *Hibiscus tiliaceus* L., silver nanoparticles were successfully fabricated [10,11]. The successful biosynthesis is due to the secondary metabolites (capping agents) presented in the extracts. Furthermore, the several bioactive components detected in the *H. tiliaceus* plant provide evidence for the antimicrobial and antioxidant properties [12,13]. The biosynthetic method by the use of plant extracts is an eco-friendly process that does not require maintaining an aseptic condition, hence this method has attracted a great deal of attention compared to chemical and physical methods, and even to the microorganisms use [3].

Silver nanoparticles (AgNPs) are increasingly used worldwide for their antimicrobial effects [14]. The AgNPs synthesized from the aqueous extract of *Cassia roxburghii* DC showed antibacterial activity against six pathogenic bacteria such as *Bacillus subtilis*, *Staphylococcus aureus*, and *Micrococcus luteus*, *Pseudomonas aeruginosa*, *Escherichia coli*, and *Enterobacter aerogenes*. In addition, the sensitivity of the strains to AgNPs was higher compared to their sensitivity to the bulk silver (AgNO_3_) [2]. Ibrahim et al. [15] observed a great potential of the biosynthesized AgNPs in the protection of rice plants from the bacterial brown stripe and infection of bacterial leaf blight. The bio-fabricated AgNPs inhibited the growth of *Staphylococcus warneri* by the cleavage of its DNA. Several other mechanisms including depletion of intracellular ATP through destabilization of the outer membrane, plasma membrane rupture, respiration blocking, and DNA damage, were proposed to be behind the antibacterial activity of silver nanoparticles [16]. On the other hand, AgNPs have a noticeable antifungal activity, Kim et al. [17] noticed that AgNPs exerted potent antifungal effects on plant pathogenic fungi through the destruction of the membrane integrity. It was shown that AgNPs inhibited the growth of several fungi species such as *Aspergillus fumigates*, *Aspergillus niger*, *Aspergillus flavus*, *Trichophyton rubrum*, *Candida albicans*, and *Penicillium* species. This inhibition was attributed to the role of AgNPs in the production of the reactive oxygen species and free radicals, which lead to protein denaturation, nucleic acid damage, lipid peroxidation, and cell wall damage [18].

The biosynthesized AgNPs were used for various applications, in the in vitro culture AgNPs were successfully applied to reduce the contaminations in the culture media [19]. Further, the addition of AgNPs in the MS medium increased the mean number of fresh shoots per explants, the percentage of explants producing shoots, and the plant survival, and this was explained by the role played by AgNPs in ethylene blockage [20]. In *Tecomella undulata*, a higher mean number of shoots per explant, mean length of shoots per explant, and percentage of explants producing shoots were detected in the explants cultured on MS basal supplemented with 60 µg/mL AgNPs compared to controls. However, media containing more than 60 µg/mL of AgNPs significantly reduced the mean length of shoots per explant. AgNPs increased explants regeneration by the diminution of the *TuACS* gene expression [21]. Sharma et al. [22] noted that the addition of Ag NPs (50 mg/L) to basal MS media improved the seedling vigor index of *Brassica juncea* by activating the antioxidant enzymes and inhibiting the content of hydrogen peroxide, malondialdehyde (MDA), and the proline content.

The genus Rumex consisted of around 200 species, one of them is *Rumex nervosus* which is widely distributed in the world. In Saudi Arabia, *R. nervosus* is a common plant in Sarawat Mountains, it is largely used as traditional herbal medicine for many diseases. The *R. nervosus* extracts showed several pharmacological effects such as antimicrobial, anti-inflammatory, antihypertensive, antidiabetic, antidiarrheal, anticoccidial, and antileishmanial activities [23,24]. An effective protocol for the propagation of *Rumex nervosus* was reported in our previous work [25]. In the current study, we first report biogenic AgNPs using silver nitrate as a precursor and the leaf extract of *Hibiscus tiliaceus* L. as a reducing and capping agent. Then, the impacts of these AgNPs to reduce the contamination in the culture media and their effects on the *R. nervosus* callus growth improvement and genetic stability were evaluated.

## 2. Results

### 2.1. Characterization of the Synthesized AgNPs

**Figure 1:**
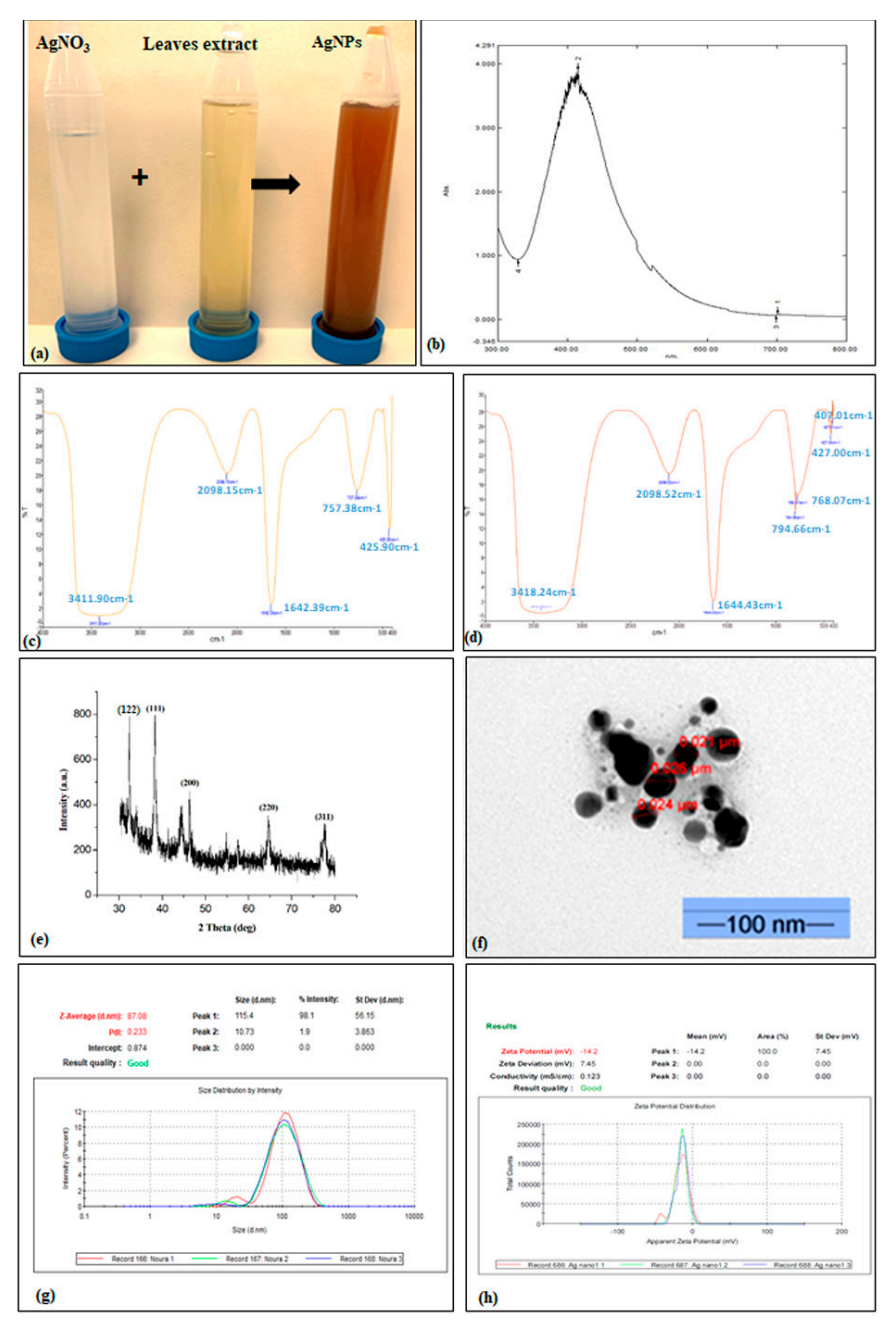
Silver nanoparticles (AgNPs) biosynthesis process (**a**), UV–visible spectra (**b**), Fourier transform infrared spectroscopy (FTIR) of the plant extract (**c**), FTIR of the fabricated AgNPs (**d**), XRD pattern of AgNPs (**e**), transmission electron microscope image (TEM) (**f**), zeta size (**g**), and zeta potential (**h**).

The fabricated AgNPs were confirmed by the change in the color of the mixture after adding the silver nitrate to the *Hibiscus tiliaceus* leaves extract. We observed that the color changed from transparent to dark brown, which revealed the transformation of the silver nitrate to the silver nanoparticles (Figure 1a). This result was further confirmed by UV–visible spectrophotometer, the estimated UV in the optical spectrum ranging from 300 to 800 nm showed maximum absorption at around 415 nm (Figure 1b). As to the FTIR, the FTIR spectrum of the plant extract showed five absorption peaks occurred at 3411.90, 2098.15, 1642.39, 757.38, and 425.90 cm^−1^, while the AgNPs displayed 3418.24, 2098.52, 1644.43, 794.66, 768.07, 427, and 407.01 cm^−1^. These absorption peaks are attributed to O-H stretching (3411.90 and 3418.24 cm^−1^), C-H stretching (2098.15 and 2098.52 cm^−1^), C=C stretching (1642.39 and 1644.43 cm^−1^), C-H bending (757.38, 768.07, and 794.66 cm^−1^), and S-S stretching (407.01, 425.90, and 427 cm^−1^) (Figure 1c,d). The XRD analysis of the synthesized AgNPs showed the presence of five intense peaks of 32,18°, 38.44°, 45.58°, 64.58°, and 77.46° at 2θ values. Compared with the standard powder diffraction card JCPD, these peaks represented (122), (111), (200), (220), and (311) plane reflections, respectively (Figure 1e). Moreover, the shape and the size of the nanosilver were detected through transmission electron microscopy (TEM). This technique showed a spherical nanoparticle with sizes between 21 and 26 nm without any agglomeration of the particles (Figure 1f). On the other hand, the zeta sizer analysis reported in the Figure 1g shows one peak with a large base indicating the variable sizes of AgNPs. Averagely, the diameter of the nanoparticles was around 87 nm. For the zeta potential, the AgNPs showed a value of −14.2 mV which proves that the surfaces of the produced nanoparticles are negatively charged (Figure 1h).

### 2.2. AgNPs Reduce the Microbial Contamination in the Culture Media

**Figure 2:**
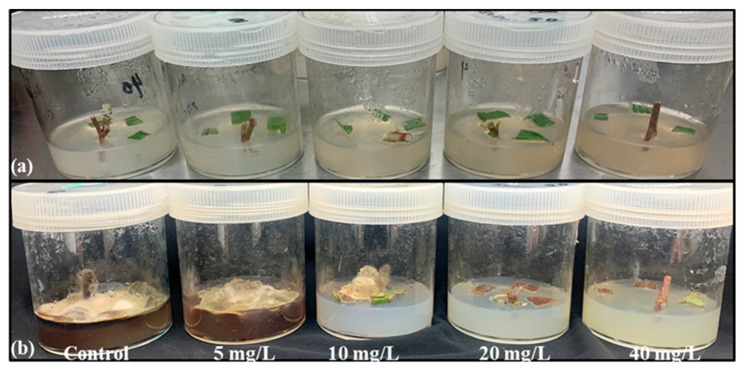
Photo showing the contamination after two weeks from the culture of *Rumex nervosus*. (**a**) At a day of culture, (**b**) after 14 days of incubation.

The effectiveness of the AgNPs to reduce the microbial contamination in the culture media was screened using *Rumex nervosus* explants cultured in MS media and supplemented with 0, 5, 10, 20, and 40 mg/L of AgNPs. After 2 weeks of incubation, we noted that the concentrations of 5 and 10 mg/L were not effective to remove the contamination from the culture media. However, the media supplemented with 20 and 40 mg/L of AgNPs showed no contamination, which suggests that these two doses could be effective for the in vitro culture medium sterilization (Figure 2).

### 2.3. Identification of the Fungus and Bacteria Isolated from the Contaminated Media

**Figure 3:**
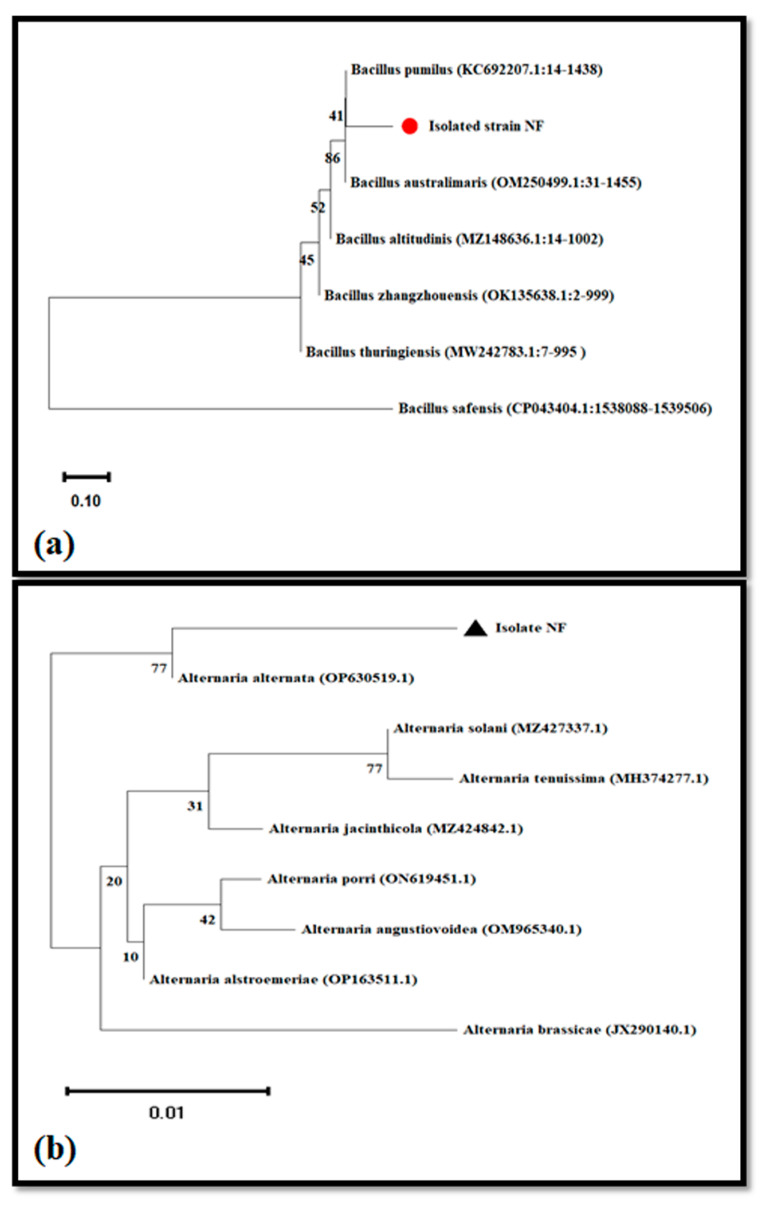
Identification of the isolated bacteria (**a**) and fungus (**b**) based on 16S rDNA and ITS rDNA sequences, respectively. Neighbor-joining phylogenetic tree was constructed using MEGA X software.

One bacteria strain and one fungal colony obtained from the contaminated jars (Figure 2), were identified on the basis of 16S and ITS rDNA, respectively, for bacteria and fungus. The amplification of the 16S was performed by 17 F (forward) and 1492 R (reverse), after editing the sequences and assembling them, the length of the sequence was approximately 1019 bp. Blasting this sequence in the NCBI GenBank revealed a similarity of 90.06% with *Bacillus pumilus*. Similarly, the phylogenetic study based on the neighbor-joining method also showed that our strain is likely *Bacillus pumilus* (Figure 3a). Regarding the internal transcribed spacer (ITS) region, it was amplified using ITS1 (forward) and ITS4 (reverse) primers. The NCBI-BLAST search of the ITS query sequence revealed a similarity of 97.63% with *Alternaria alternata* (OP630519.1). Moreover, the phylogenetic tree constructed by the neighbor-joining method indicated that our isolate is closely related to *Alternaria alternata* more than the other genus of Alternaria (Figure 3b).

### 2.4. Quantification of the Antifungal and Antibacterial Effects of AgNPs

**Figure 4:**
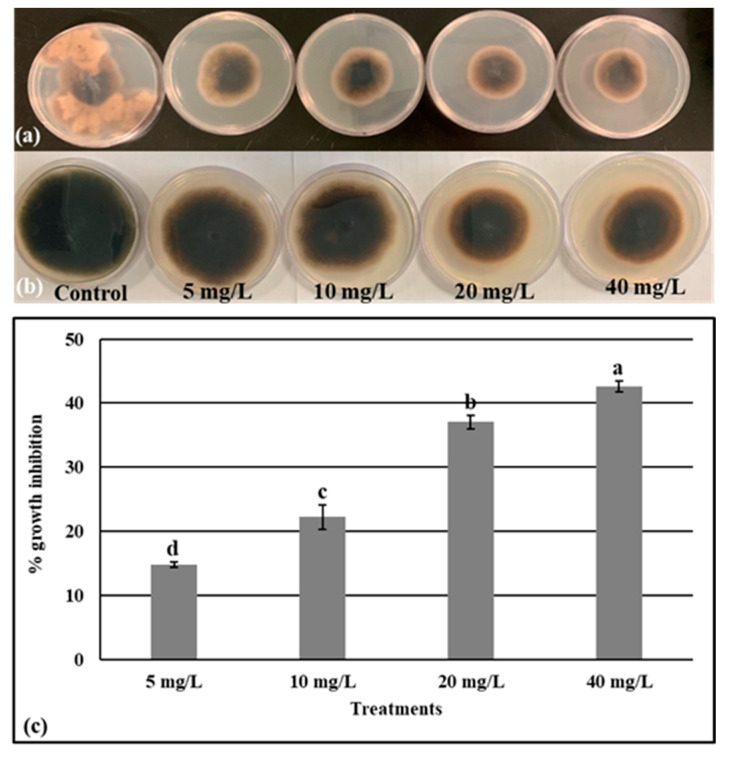
Morphology of the isolated fungus after 3 days (**a**) and 1 week (**b**) of incubation in MS supplemented or not with AgNPs. (**c**) The percentage of growth inhibition of the isolated fungus after 7 days of exposure to AgNPs. Values are the means of three values ± SD, different letters on bars indicate the significant differences according to Duncan’s test (*p* < 0.05).

**Figure 5:**
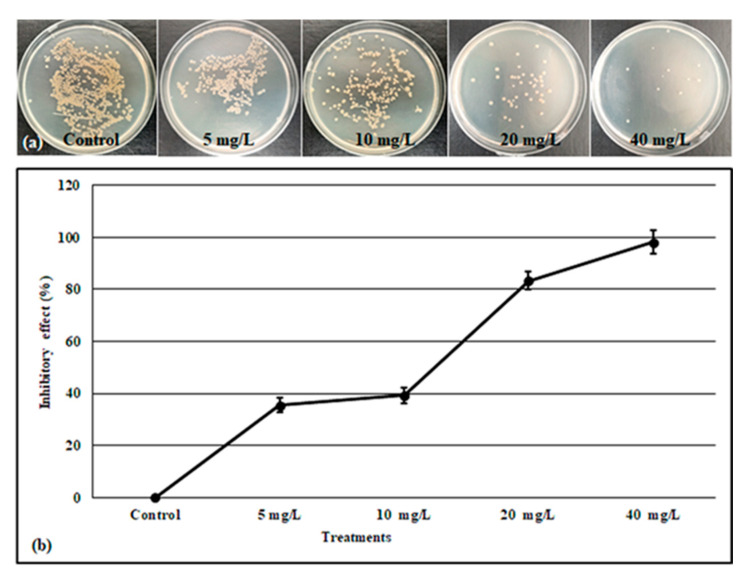
Photo of isolated bacteria after 24 h of incubation under different concentrations of AgNPs (**a**), inhibitory effect of different concentrations of AgNPs (**b**). Values are the means of three values ± SD, the significant differences according to Duncan’s test (*p* < 0.05) are indicated by different letters.

The fungus isolated from the contaminated jars and identified as described above was selected to quantify the antifungal effect of the fabricated AgNPs. The mycelium (*Alternaria alternata*) growth under different concentrations of AgNPs was measured on PDA (Figure 4a,b). The result of this test showed that the maximum antifungal activity was attained by an increment of AgNPs doses. In comparison to the control (without nanoparticles), the mycelium growth was inhibited by 14.8%, 22.2%, 37.03%, and 42.59%, respectively, under 5, 10, 20, and 40 mg/L of AgNPs (Figure 4c). On the other hand, the antibacterial activity of the biogenic AgNPs toward *B. pumilus* isolated from the contaminated media was also analyzed. Figure 5 shows that the *B. pumilus* is significantly inhibited by 35.41, 39.29, 83.37, and 98.12 %, respectively, under 5, 10, 20, and 40 mg/L of AgNPs when compared to the control.

### 2.5. Effect of AgNPs on Callus Growth and Its Genetic Stability

**Figure 6:**
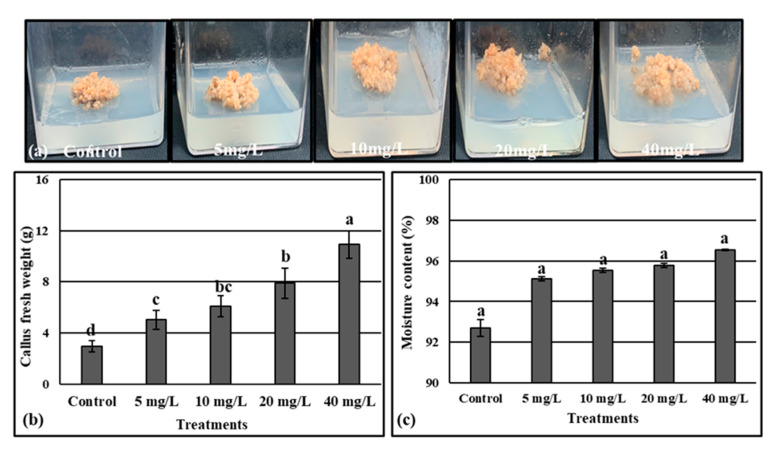
Effect of different concentrations of AgNPs on callus morphology of *Rumex nervosus* (**a**), callus fresh weight (**b**), and callus moisture content (**c**). Values are the means of three values ± SD, different letters on bars indicate the significant differences according to Duncan’s test (*p* < 0.05).

The callus induction from the medicinal plant *Rumex nervosus* was best shown in Murashige and Skoog medium supplemented with 2.5 μM of 2,4-dichlorophenoxyacetic acid and 0.1 μM of BAP [25]. Synergistically with these MS ingredients, different concentrations of AgNPs (5, 10, 20, and 40 mg/L) were employed (Figure 6a). After one month of incubation, we observed that the supplementation of the media with the AgNPs significantly increased the callus fresh weight. Moreover, the supplementation with 40 mg/L exhibited higher callus fresh weight compared to the control and to the other treatments (Figure 6b). Nevertheless, for the callus moisture content, no significant effect of AgNPs treatments was registered (Figure 6c).

**Figure 7:**
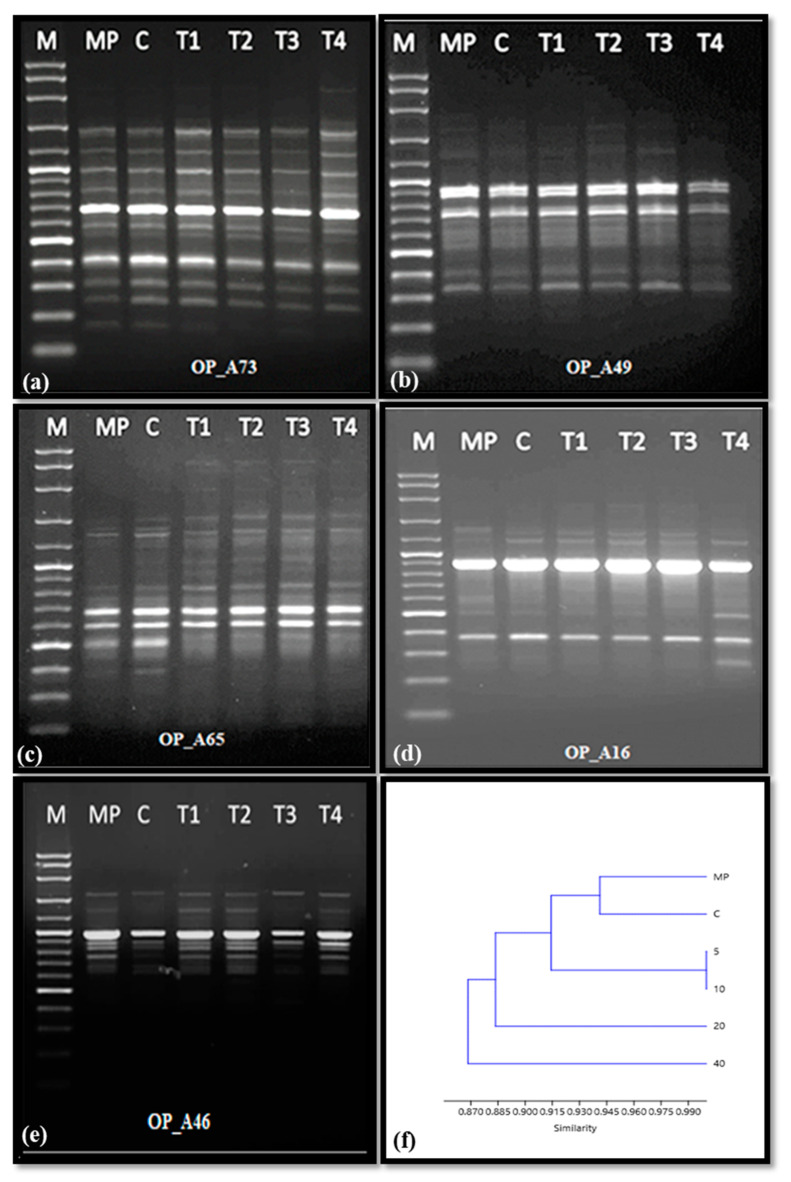
Amplification profiles of RAPD primers OP_A73 (**a**), OP_A49 (**b**), OP_A65 (**c**), OP_A16 (**d**), OP_A46) (**e**), and the UPGMA analysis dendrogram showing the relationships between the treatments (**f**). DNA ladder (M), Mother plant (MP), control callus (C), T1, T2, T3, and T4 represented the callus of *Rumex nervosus* treated with 5,10, 20, and 40 mg/L of AgNPs, respectively.

The genetic uniformity of callus treated with different doses of AgNPs was analyzed using RAPD markers. Among 10 tested primers (only 5 primers (OP_A73, OP_A49, OP_A65, OP_A16, and OP_A46) were selected owing to their abilities to produce clear and scorable bands (Figure 7). The 5 RAPD primers generated around 40 bands ranging from 200 to 1600 bp, further, the number of the unique bands were 12, 6, 9, 7, and 6, respectively, for OP_A73, OP_A49, OP_A65, OP_A16, and OP_A46 primers. Among the total recorded bands, 35 bands were monomorphic and 5 were polymorphic representing 87.5% and 12.5%, respectively. The polymorphism was detected by the loci appearing and disappearing in the AgNPs-treated callus, and this was more observed in the callus treated with 40 mg/L of AgNPs. Additionally, the UPGMA dendrogram based on the Jaccard similarity index analysis showed a similarity of 87.1% amongst the Ag NPs treated callus, control callus, and the mother plant (Figure 7f).

## 3. Discussion

The metallic nanoparticles are fabricated by different methods such as chemical and physical methods. However, these methods are energy consumers, toxic, expensive, and not suitable for biological applications [26]. Recently, there is an increasing demand for metallic nanomaterials by adopting green and environmental methods to avoid health and environmental risks. Among all known metallic nanomaterials, the green synthesis of AgNPs using plant extracts as an alternative stabilizer has gained increasing interest [11]. In this study, the AgNPs were biologically synthesized using the aqueous leaf extract of *Hibiscus tiliaceus*, which plays the role of the reducing and capping agent. Konduri et al. [27] and Rani et al. [10] reported that the *H. tiliaceus* extract was successfully used to reduce and stabilize the ZnONPs and AgNPs. The GC-MS analysis reported by [12] showed the presence of 23 phytochemical components in leaf extracts of *H. tiliaceus* which could be behind its potential as a capping and reducing agent in the green synthesis of AgNPs. After adding the aqueous leaf extracts of *H. tiliaceus* to the silver nitrate solution, we observed a change in color from light yellowish to dark brown. This change in color is attributed to the excitation of surface plasmon vibration in the AgNPs. Moreover, the color change in the reaction mixture is an indicator of the reduction of the ion Ag+ to Ag° in the silver nitrate solution, which proves the formation of AgNPs [28]. Kahsay et al. 2018 [29] reported that the mechanism of bio-reduction of Ag NPs using plant extract consists of three stages which start with the reduction of Ag^+^ during the activation phase, followed by nucleation of the reduced silver atom. However, in the third stage (growth stage) the formation of the final shape of the Ag NPs occurs. The nanostructures for the prepared AgNPs were then confirmed by UV–vis absorption, FTIR, XRD, TEM, zeta size, and zeta potential. The examination with the UV spectrophotometry showed a peak at 415 nm (Figure 1b), confirming the presence of AgNPs in the reaction mixture. This is in agreement with the result of [30] who observed that the UV–vis spectra analysis of the silver nanoparticles biosynthesized using *Citrobacter* spp. MS5., revealed an absorption at 415 owing to the surface resonance. The FTIR analysis showed that the absorption peaks detected for the plant extract are slightly changed in comparison with the absorption peaks of AgNPs which indicate the presence of capping and stabilizing biomolecules with the nanoparticles. The functional groups corresponding to the absorption peaks are assigned to the alcohol and phenols of –OH stretching, hydrocarbon (C–H) stretching vibration, -C=C- stretching of alkenes, cis-C-H out-of-plane bend, and aryl disulfides S-S stretch, respectively, for (3411.90 and 3418.24 cm^−1^), (2098.15 and 2098.52 cm^−1^), (1642.39 and 1644.43 cm^−1^), (700 cm^−1^ broad), and (407.01, 425.90 and 427 cm^−1^) bands [31,32,33,34,35]. The XRD diffractogram (Figure 1e) showed five peaks of 32.18°, 38.44°, 45.58°, 64.58°, and 77.46° at 2θ values corresponding to the (hkl) values of (122), (111), (200), (220), and (311), respectively, as compared with the standard powder diffraction card of JCPDS, silver file No. 04-0783 [36,37,38,39,40]. However, the non-assigned peaks presented in the XRD diffractogram may be due to the presence of biological capping (phytochemicals of the plant extract) on the surface of AgNPs [41]. Further, the shape and the particle size of the biosynthesized AgNPs were confirmed by the analysis of the transmission electron microscopy (TEM) technique. The TEM image shows that the AgNPs have a spherical shape with a size ranging from 21 to 26 nm. The result obtained by [42] showed that the AgNPs biologically synthesized using *Ochradenus arabicus* extract were spherical in shape with a size ranging between 9 and 30 nm. The nanoparticles’ size depends on the plant species and the source of extracts that are used as reducing and capping agents. Moreover, the size distribution of nanoparticles depends on the relative rate of nucleation and the extent of agglomeration [43,44]. Additionally, an optimal size of the nanoparticles can be achieved by adjusting the extracted quantity, temperature, and metal ion concentration [45,46]. Using the zeta size technique (Figure 1g), we noted that the particle sizes were different from that measured by TEM. This variation is due because zeta size analysis determines the hydrodynamic size, while the TEM technique shows only the metallic core. Furthermore, TEM gives the selected area electron diffraction (SAED) patterns which reveal the distribution and crystalline nature of particles in the focusing zone [47]. On the basis of zeta potential measurement, the zeta potential value of the synthesized AgNPs is −14.2 mV. The zeta potential is used as an indicator to understand the stability of nanoparticles in the aqueous suspensions. Indeed, the positive or negative charge on the surface of nanoparticles provides stability and prevents the aggregation of the nanoparticles by pushing the same charges [48].

Plant micropropagation is challenged by microbial contamination, which is a main problem for plant tissue culture. The use of antibiotics has harmful effects on plants, and their continued use makes bacteria more resistant. Furthermore, the chemicals used to avoid the contaminations are toxic to the explant and have limited effectiveness. Nanobiotechnology offers an alternative approach to dealing with bacterial and fungal contamination [49]. Arab et al. [50] noted that using nanosilver in a culture medium after surface sterilization is effective in removing fungal and bacterial contaminations. Our findings showed that AgNPs at the level of 20 and 40 mg L^−1^ had a high potential to eliminate the microbial contamination from the culture medium, however, the low dose (5 mg L^−1^) had no effect in the inhibition of the microbial contaminants (Figure 2). Similarly, the result data obtained by [19] showed that the supplementation of the culture media with AgNPs allowed for the obtaining of a higher number of sterilized explants. In the next step, the antifungal and antibacterial potency were investigated against *Alternaria alternata* and *Bacillus pumilus* (Figure 4 and Figure 5). These two strains were isolated from the contaminated media without or with low doses of AgNPs (5 and 10 mg/L), and identified using 16S and ITS primers, as these two genetic markers are commonly chosen for the molecular identification of bacteria and fungi [51,52]. Our study demonstrated that the biosynthesized AgNPs significantly reduce the growth of *Alternaria alternata*, and more reduction was registered in the media supplemented with 20 and 40 mg/L. The biosynthesized AgNPs using plant leaf extract had broad-spectrum antifungal activity against several fungi such as *Gloeophyllum abietinum*, *G.trabeum*, *Chaetomium globosum*, and *Phanerochaete sordida* [53]. The AgNPs’ antifungal potential is affected by various physicochemical parameters including the shape, size, surface charge, concentration, and colloidal state [54]. In this context, the result obtained by [55] showed that the increment in the concentration of AgNPs and the decrement of its nanosize resulted in a higher antifungal activity against the plant pathogenic *Fusarium oxysporum*. The antifungal role played by the AgNPs may be attributed to the production of reactive oxygen species (ROS), which leads to cell wall disintegration, surface protein damage, nucleic acid damage, blockage of proton pumps, and thereby cell apoptosis [18]. Darwesh and Elshahawy [56] reported that the biosynthesized AgNPs had a promising antifungal activity against *Stromatinia cepivora* by inactivating the sclerotial formation.

Concerning the effect of AgNPs on the growth of isolated bacteria *B. pumilus*, we observed that the AgNPs effectively inhibited the bacteria growth at doses of 40 mg/L. Moreover, the antibacterial activity increased with an increase in the concentration of Ag NPs, which indicates that the response of the bacteria depends on the used dose [33]. The AgNPs have an antibacterial powerful toward Gram-positive and Gram-negative bacteria through multifaceted mechanisms [57]. However, the silver ions (Ag^+^) released from the AgNPs, may be considered as one of the mechanisms behind the bactericidal activity of AgNPs, since these released ions can deactivate the enzyme for cell respiration and replication inducing thus the death of the cell. [58,59]

In addition to its benefit in the elimination of microbial contaminants, the application of nanoparticles (NPs) demonstrated a positive role in callus induction, organogenesis, somatic embryogenesis, somaclonal variation, genetic transformation, and secondary metabolite production [60]. In this study, we recorded a significant callus growth by adding AgNPs in the culture media, further, 40 mg/L exhibited higher callus fresh weight than that obtained from the control and other treatments (Figure 6). The application of AgNPs in tissue culture medium promoted callus induction frequency, callus regeneration, and rhizogenesis of Indica rice in a dose-dependent manner [61]. Likewise, the in vitro supplementation by AgNPs positively affects the callus induction and growth in *Caralluma tuberculate* [62]. In the closed containers used for the in vitro culture, the produced ethylene can severely influence the cultured explants. However, this ethylene influence can be suppressed by AgNPs which are considered an ethylene suppressor by downing the regulation of the genes related to the biosynthesis of this hormone [61].

The effect of AgNPs on the genetic stability of the callus exposure to the treatment of 0, 5, 10, 20, and 40 mg/L AgNPs was performed by using RAPD markers. Among the treatments, 12.5% of polymorphism was detected (Figure 7). The polymorphism owing to the presence or absence of DNA loci between the treatments may be attributed to DNA damage by AgNPs which can interact with the phosphorus of DNA molecules, thus causing a DNA mutation [63]. In addition, it was reported that nanoparticles can bind to nucleic acids, which leads to a change in the DNA helix conformation, and thereby, change the nitrogenous bases’ orientation in the DNA strand [64]. Based on RAPD-PCR analysis, it was shown that AgNPs at a dose of 25, 50, and 75 ppm caused a reduction in the genomic template stability percentage values, and this was reflected by the changes in bands number as well as the band’s intensity [65]. The RAPD markers are considered an inexpensive and effective tool to easily distinguish genetic variation. In in vitro propagated chrysanthemums treated with different concentrations of AgNPs, RAPD markers were successfully employed to detect the polymorphic loci [66,67].

## 4. Materials and Methods

### 4.1. Plant Materials

The leaves of *Hibiscus tiliaceus* plant were collected from the garden of King Saud University, Riyadh, Kingdom of Saudi Arabia, and confirmed through the specimen (24595) deposited at the herbarium of the botany department, College of Science, King Saud University.

The *Rumex nervosus* was collected from Wadi Ghazal in the city of Taif, Kingdom of Saudi Arabia. This plant was molecularly identified (using the ITS1 forward and the ITS4 markers) and deposited in the NCBI under the accession number JX026929.

### 4.2. Extract Preparation and Nanoparticles (AgNPs) Synthesis

The leaves of *H. tiliaceus* were washed, cut into small pieces, and dried at room temperature for two weeks. The dried materials were ground to a fine powder, and then 5 g of leaf powder was mixed with 100 mL of milli Q water and kept for shaking for 12 h. Then after, the extract was filtered through Whatman filter paper no. 40 and stored at 4 °C for further use.

For the biosynthesis of silver nanoparticles (AgNPs), 10 mL of aqueous leaf extract was added to 190 mL of 1 mM AgNO_3_ in Erlenmeyer flask and incubated for 3 h in the rotary shaker (New Brunswick InnovaR 44 Incubator Shaker, Methuen, MA, USA). The change in color to dark brownish was considered to be the indicator of AgNPs formation. The synthesized nanoparticles (AgNPs) were kept at 4 °C for the characterization and the bioactivity study.

### 4.3. Characterization of AgNPs

The biosynthesis of silver nanoparticles was first monitored using UV–visible (UV–vis) spectrophotometer (Shimadzu UV-1800, Kyoto, Japan) in the range of 300 to 800 nm. For the Fourier transform infra-red spectra (FTIR) of silver nanoparticles, sample was pelletized with potassium bromide (KBr) and then analyzed in Fourier transform infrared spectrophotometer (8400S, Shimadzu, Japan) in the range of 4000–500 cm^−1^. The AgNPs X-ray diffraction (XRD) measurement was performed using the Bruker D8 Discover XRD system in 2θ range of 30–80°. The obtained XRD values were analyzed with OriginPro software 2021, and then compared with the standard powder diffraction card JCPDS) File No.: 04-0783 corresponding to silver. The size and the shape of AgNPs were characterized by using transmission electron microscopy (TEM) (JEM-1011; JEOL Ltd., Tokyo, Japan). For that, the aqueous AgNPs were dried on the carbon-coated copper TEM grids for 5 min, and then the TEM was performed at an accelerating voltage of 80 Kv. The particle size distribution and the zeta potential were determined by the use of Malvern Zetasizer machine (Malvern Instruments Ltd., Malvern, UK).

### 4.4. Effect of AgNPs on Reducing the Contamination of the Culture Media

The leaves and stems of the *Rumex nervosus* were cut into small pieces and washed under running tap water for 30 min. In the laminar flow chamber, the explants were disinfected with 50% of commercial Clorox for 10 min, followed by several washes with sterilized distilled water to remove the traces of bleach. Then the explants were transferred to Murashige and Skoog media (MS) [68] containing 20 g/L sucrose and supplemented with five doses of silver nanoparticles (0, 5, 10, 20, and 40 mg/L). After two weeks of incubation at 16/8 h light/dark photoperiod and at a temperature of 25 °C, the result of this experiment was photographed to document the presence and the absence of microbial contamination in the jars.

### 4.5. Isolation and Identification of the Microbial Contaminants

To quantify the antifungal and the antibacterial activities of the fabricated AgNPs, one single fungus and bacteria were isolated from the contaminated jars (described above) and identified as follows: 

The fungus was picked from the contaminated media and transferred to a new Petri dish containing potato dextrose agar (PDA). A series of sub-culturing was performed in order to obtain pure colony. The one-week-old fungus was used for the DNA extraction following the manufacturer’s instructions for the DNeasy Plant Mini Kit (Qiagen, Hilden, Germany). Then after, the amplification of the ITS region (the internal transcribed spacer) was performed by the universal primers ITS1 (forward) and ITS4 (reverse). The PCR reaction was carried out in GE Healthcare PCR beads (illustra™puReTaq Ready-To-Go PCR Beads, Little Chalfont, Amersham place, UK) to which we added 20 µL of deionized water, 0.5 µL from forward and reverse primer, and 2 µL of the genomic DNA. The Applied Biosystems Thermal Cycler was used to perform the PCR reaction under the following conditions: one cycle at 94 °C for 5 min; 30 cycles at 94 °C for 1 min, annealing at 55 °C for 1 min, extension at 72 °C for 1 min; and a final extension step of 72 °C for 5 min.

For the isolated bacteria (one morphologically distinct colony), the DNA extraction was performed by the use of GenElute™ Bacterial Genomic DNA Kits (Sigma-Aldrich, St. Louis, MO, USA) following the manufacturer’s instructions. The DNA quantity and integrity were checked in NanoDrop™ 8000 Spectrophotometer (Thermo Scientific, Wilmington, NC, USA) and 1% agarose gel. Then after, the 16S region was amplified using the universal primer 17 F (forward) and 1492 R (reverse). The PCR reaction consisted of GE Healthcare PCR beads (illustra™puReTaq Ready-To-Go PCR Beads), 20 µL of deionized water, 0.5 µL from forward and reverse primer, and 2 µL of the genomic DNA. The amplification was performed following the program described by Thomas (2004) [69] with few modifications: 95 °C for 5 min followed by 30 amplification cycles of 94 °C for 30 s, 50 °C for 30 s, and 72 °C for 30 s and a final extension step of 72 °C for 5 min.

After checking the amplified fragments on 1.5% agarose gel, the PCR products were purified and sent to Macrogen Inc. (Seoul, Republic of Korea) for sequencing. 

### 4.6. AgNPs Antifungal Activity

The isolated fungus (described above) was incubated on potato dextrose agar (PDA) for one week at 27 °C. Using sterile cork-borer (4 mm diameter), plugs from the edge of this colony were taken and inoculated in the center of petri plates containing MS medium with different concentrations of AgNPs (0, 5, 10, 20, and 40 mg/L). The petri plates (three replications for each treatment) were sealed carefully and incubated for 7 days at 27 °C. Then, the antifungal activity of the green AgNPs was recorded by measuring the percentage growth inhibition (PGI) as described in [70]. The PGI (%) = [(GC − GT)/GC] × 100, where GC is the growth of the mycelium in the control plates and GT is the growth of the mycelium in the treated plates. 

### 4.7. AgNPs Antibacterial Activity

The isolated bacteria described above were inoculated in 20 mL of Luria broth (LB) and incubated overnight at 37 °C, 150 rpm. The OD600 was measured and then 100 μL of this broth solution (10^6^ CFU/mL) was transferred to new tubes containing 20 mL LB and supplemented with the various concentration of AgNPs (0, 5, 10, 20, 40 mg/L), after incubation at 37 °C for 12 h. The OD600 of each tube was taken and 100 μL of the suspension bacteria was cultured in LB agar media. This experiment was carried out in triplicate for each treatment, the colony viability was estimated by counting, and the inhibitory effect percentage of the AgNPs was calculated using the formula reported by [71].

Inhibitory effect (%) = (CFU_control_ − CFU_treatment_)/CFU_control_ × 100%,

where CFU_control_ is the colony forming unit in the sample without AgNPs and CFU_treatment_ is the colony forming unit in the sample containing AgNPs.

### 4.8. Effect of AgNPs on Callus Growth Induced from Rumex nervosus Explants

The leaves and stems of the *Rumex nervosus* were cut into small pieces and washed under running tap water for 30 min. In the laminar flow chamber, the explants were disinfected with 50% of commercial Clorox for 10 min, followed by several washes with sterilized distilled water to remove the traces of bleach. Then, the explants were transferred to solid MS media (0.7% agar) containing 30 g/L sucrose supplemented with 2.5 µM of 2,4-dichlorophenoxyacetic acid (2,4-D) and 0.1 µM of Benzylaminopurine (BAP) as described in [25] and enriched with different levels of AgNPs (5, 10, 20, 25, and 40 mL/L). The jars were maintained in the growth chamber at 25 ± 2 °C and a photoperiod of 16/8 h (light/dark) for one month. Then, the callus growth vigorous was noticed by the measure of the fresh weight and the moisture content.

### 4.9. Genetic Stability of the Callus

For studying the genetic stability after the treatments with AgNPs, the genomic DNA was extracted from 100 mg of callus (1-month-old) following the manufacturer’s instructions of the DNeasy Plant Mini Kit (Qiagen). The quality of the DNA was checked by running it on 1% agarose gel electrophoresis, while for the DNA quantity, a Nanodrop 8000 spectrophotometer (Thermo Scientific) was used. The PCR amplification was performed in a reaction volume of 20 µL containing 4 µL of PCR Master Mix 5X (Thermo Scientific), 2 µL of RAPD primers as listed in Table 1, 2 µL of the genomic DNA, and 12 µL of milli-Q water. The PCR reaction was set in Veriti 96-well Thermal Cycler (Applied Biosystem, Singapore) as the following program: 94 °C for 5 min for one cycle, 35 cycles at 94 °C at 1 min, annealing temperature at 36 °C for 1 min, 72 °C for 1.5 min for complementary strands synthesis, and final extension at 72 °C for 5 min. The amplified DNA was separated by running on 1.2% gel agarose and then photographed with Syngene gel documentation system. The genetic stability of the samples was recorded based on the number and location of the bands, knowing that smeared and unclear bands were not accounted for.

### 4.10. Statistical Analysis

For the statistical analysis, we used the software IBM-SPSS version 25. The data (three replicates) were applied to one-way ANOVA and then analyzed with Duncan’s test. The significant difference at *p* ≤ 0.05 was indicated by the different letters on the chart bars.

The isolated fungus and bacteria were identified through the NCBI-BLAST search. Next, the neighbor-joining method by bootstrapping 1000 times in MEGA X software version 25 was performed to construct the phylogenetic tree of the two strains.

## 5. Conclusions

In conclusion, this study reports the biogenic AgNPs using the aqueous leaf extract of *Hibiscus tiliaceus*. The nanostructures that were efficient for the green AgNPs were confirmed by UV–vis absorption, FTIR, XRD, TEM, zeta size, and zeta potential techniques. The application of AgNPs to the culture medium displayed a high potential to eliminate microbial contamination, this was concretized by the significant antibacterial and antifungal activity against *Bacillus pumilus* and *Alternaria alternata*, respectively. Additionally, the AgNPs supplementation resulted in an increase in the fresh weight of callus induced from the *Rumex nervosus*. Moreover, callus exposed to AgNPs showed a genetic variation of 12.5% which was reflected by the bands appearing and disappearing in the electrophoresis gel following an RAPD PCR amplification. This attempt to show the effectiveness of AgNPs (especially at 20 and 40 mg/L) on microbial contamination reduction as well as the callus growth improvement could be followed by further molecular research to understand the mechanism behind the role of the biosynthesized AgNPs.

## Figures and Tables

**Table 1:** The primer sequences used for the PCR.

Primer	Sequence (5′--------- 3′)
ITS1-F	TCCGTAGGTGAACCTGCG
ITS4-R	TCCTCCGCTTATTGATATGC
27-F	AGAGTTTGATCCTGGCTCAG
1492-R	GGTTACCTTGTTACGACTT
OP-A16	AGCCAGCGAA
OP-A46	GAACGGACTC
OP-A49	CTCACCGTCC
OP-A65	TGAGCGGACA
OP-A73	GGGGTGACGA

## Data Availability

Data are contained within the article.

## Sample Availability

Samples of the compounds are available from the authors.
